# Invasive Infections Caused by *Nannizziopsis* spp. Molds in Immunocompromised Patients 

**DOI:** 10.3201/eid2403.170772

**Published:** 2018-03

**Authors:** Céline Nourrisson, Magali Vidal-Roux, Sophie Cayot, Christine Jacomet, Charlotte Bothorel, Albane Ledoux-Pilon, Fanny Anthony-Moumouni, Olivier Lesens, Philippe Poirier

**Affiliations:** Centre Hospitalier Universitaire Clermont-Ferrand, Clermont-Ferrand, France; 1 These authors contributed equally to this article.

**Keywords:** *Nannizziopsis*, invasive fungal infection, central nervous system fungal infection, opportunistic fungal pathogen, emerging disease, fungi, molds, immunocompromised patients

## Abstract

We report 2 new cases of invasive infections caused by *Nannizziopsis* spp. molds in France. Both patients had cerebral abscesses and were immunocompromised. Both patients had recently spent time in Africa.

*Nannizziopsis* spp. molds have been reported in extremely rare cerebral and disseminated infections (*1**,**2*), (Table). We describe 2 cases of *Nannizziopsis* infection diagnosed in France during the past 2 years. Both case-patients were immunocompromised and had recently returned from Africa.

**Table:** Characteristics of *Nannizziopsis* sp. infection in humans*

Year (reference)	Age, y/sex	Country	Underlying condition or context	Species	Localization	Positive samples	Treatment	Outcome
2017 (this study)	52/F	France	HIV, living in Mali	*Nannizziopsis* sp.	Brain abscess	Cerebral biopsy, CSF	AmpB for 1 mo, then VCZ	Recovery but neurologic sequela after 2 mo
2015 (this study)	63/F	France	Leukemia, recent travel to Senegal	*N. obscura*	Brain abscess	Blood culture, CSF, ascites fluid	Not treated (death before diagnosis)	Death before diagnosis
2005 (*2*)	38/M	Germany	HIV, travel to Nigeria	*N. obscura*	Brain abscess	Needle aspiration of brain lesion	VCZ	Recovery without sequelae after 4 mo
2005 (*5*)	40/M	United States	HIV	*N. infrequens*	Lung	Bronchial washing	Not treated, considered as a contaminant	Recovery after treatment of CMV infection
2000 (*3,4*)	32/M	United States	Travel to Nigeria	*N. hominis*	Lymph nodes, heart, lungs, spleen, kidneys	3 lymph nodes	ITRA for 2 y	NA
1994 (*3*)	NA/M	United States	HIV	*N. hominis*	Right thigh mass	Deep muscle mass on the right thigh, right groin, buttock, and lung	ITRA	Death after 8 mo
1982 (*1*)	24/M	United States	Travel to Africa	*N. obscura*	Abscess in right ankle, osteomyelitis	2 biopsies of abscess in tibia	AmpB for 4 mo	Recovery after 4 mo

*AmpB, amphotericin B; CMV, cytomegalovirus; CSF, cerebrospinal fluid; ITRA, itraconazole; NA, not available; VCZ, voriconazole.

## The Cases

Case-patient 1 was a 63-year-old woman from France who had been treated for T-cell prolymphocytic leukemia diagnosed in December 2014. She initially received alemtuzumab, switching to bendamustine in March 2015 after tumor progression. That treatment failed, and idelalisib was started on July 11, 2015. The patient was hospitalized 2 days later for acute renal failure with mild fever. She became confused and drowsy, and cerebral computed tomography (CT) scan showed systematized subcortical hypodense areas. Lumbar puncture showed neoplastic cells in the cerebrospinal fluid (CSF) and glucose concentration within reference range. Bacteriological and fungal cultures were sterile. A large volume of ascites fluid remained, despite iterative punctures with negative bacteriological cultures. We initiated intrathecal chemotherapy with methotrexate/cytarabine/methylprednisolone. However, the patient’s condition worsened, with heart failure and loss of consciousness. On July 18, we took new specimens of CSF, bronchial wash, ascites fluid, and blood cultures and sent them for bacteriological investigation. We started empiric treatment with imipenem/aminoglycosides, but the patient died on July 19 of septic shock. No autopsy was performed. Extended-spectrum β-lactamase–producing *Escherichia coli* sensitive to imipenem grew quickly in 1 pair of blood cultures. A second pair was positive 4 days later, with the presence of large septate fungal hyphae and arthroconidia. White and thin cottony mold colonies grew on Sabouraud media incubated at 35°C (Technical Appendix Figure 1). We performed best model determination and phylogenetic analyses in MEGA6 (http://www.megasoftware.net). We identified *N. obscura* by sequencing the 18S-internal transcribed spacer (ITS) 1–5.8S-ITS2 region (Technical Appendix Figure 2). The strain had low MICs for antifungals as defined by the European Committee on Antimicrobial Susceptibility Testing (http://www.eucast.org/): amphotericin B 0.06 µg/mL, itraconazole 0.25 µg/mL, voriconazole 0.03 µg/mL, posaconazole 0.06 µg/mL, caspofungin 0.5 µg/mL, and micafungin 0.015 µg/mL. We performed mycologic investigations of CSF and ascites fluid a posteriori on frozen aliquots and conducted PCR assays targeting the ITS region on CSF sampled on July 15 and July 19 and on ascites fluid sampled on July 19. We observed positive amplifications in all samples; subsequent sequencing confirmed the presence of DNA from *N. obscura*. We investigated the origin of the patient’s contamination. She had made several trips to Senegal, the latest in January 2015, during which an ulcerative inflammatory lesion developed on her left little finger. However, Grocott stain and PCR on paraffin-embedded tissue of skin biopsy were negative, and we attributed the lesion to the hematological malignancy.

Case-patient 2 was a 52-year-old woman from France living in Mali, who was hospitalized in Bamako in November 2016 for cough, fever, alteration of general state, and headache. She tested seropositive for HIV (CD4 3/µL; HIV-1 viral load 45.300 copies/mL). Chest radiograph showed bilateral pneumonia, and cerebral CT scan showed a single process on the left temporal lobe. Antiretroviral therapy was initiated with a combination of efavirenz/lamivudine/tenofovir associated with isoniazid, metronidazole, amoxicillin/clavulanate, and trimethoprime/sulfamethoxazole. Because of worsening of her neurologic status, she was repatriated to France. At hospital admission on January 12, 2017, she had a lesion on the left middle fingernail suggestive of onychomycosis, hemiparesis, and paralysis of the right side of the face associated with Broca’s aphasia. A thoracic-abdominal-pelvic scan revealed a nodular lesion in the right lung (Figure, panel A) and multiple partly calcified pleural lesions. Bacteriological assays, including investigation for mycobacteria on bronchoalveolar lavage (BAL) fluid, showed negative results. A *Penicillium* grew rapidly on mycological medium, and *Pneumocystis jiroveci* PCR was slightly positive. Cranial tomodensitometry showed multiple gadolinium-enhancing nodules surrounded by edema (Figure, panel B). We initiated fluconazole and pyrimethamine/sulfadiazine and switched antiretroviral therapy to raltegravir/abacavir/lamivudine after the onset of acute renal insufficiency. Hemiplegia developed 15 days later. A new CT scan showed stable cerebral lesions but an increase in surrounding edema. We performed a lumbar puncture and started intravenous corticotherapy. CSF contained 127 leukocytes (61% lymphocytes) and showed hypoglycorrhachia. Investigations for toxoplasmosis, cryptococcosis, histoplasmosis, tuberculosis, and CMV showed negative results, but ITS-targeting PCR results were positive on CSF. The sequence was closely related to *N. obscura*, but mycological cultures were sterile (Technical Appendix Figure 2). The result of a β-D-glucan assay of serum was positive (983 pg/mL; Fungitell, Associates of Cape Cod, Inc., http://www.acciusa.com/clinical/fungitell/index.html) and galactomannan antigen was negative. On February 6, we performed a cerebral biopsy. Histopathological examination showed granuloma containing hyphae (Figure, panels C, D), and on the fourth day of incubation, white mold grew on Sabouraud media at 25°C and 35°C (Technical Appendix Figure 1). The phylogenetic analyses of the 18S-ITS1–5.8S-ITS2 region confirmed that the fungus belonged to *Nannizziopsis* spp. Although the 18S region was closely related to *N. obscura*, the ITS1 region had a large insertion, suggesting the strain does not belong to the described *Nannizziopsis* species (Technical Appendix Figure 2). Strain MICs as defined by the European Committee on Antimicrobial Susceptibility Testing were amphotericin B 0.25 µg/mL, itraconazole 0.03 µg/mL, voriconazole 0.125 µg/mL, posaconazole 0.25 µg/mL, caspofungin 0.25 µg/mL, and micafungin <0.008 µg/mL. We initiated liposomal amphotericin B (5 mg/kg). On July 16, the patient became drowsy with a bilateral pyramidal syndrome and moderate reactive mydriasis. Cranial CT scan showed an increase in the abscesses and edema with brain displacement. We performed a craniotomy. One month after diagnosis, the patient’s general status had improved, with regression of the lung lesions and cerebral abscesses; her CD_4_ cell count was 50/µL and HIV-1 load <40 copies/mL. We switched her antifungal treatment to voriconazole.

**Figure:**
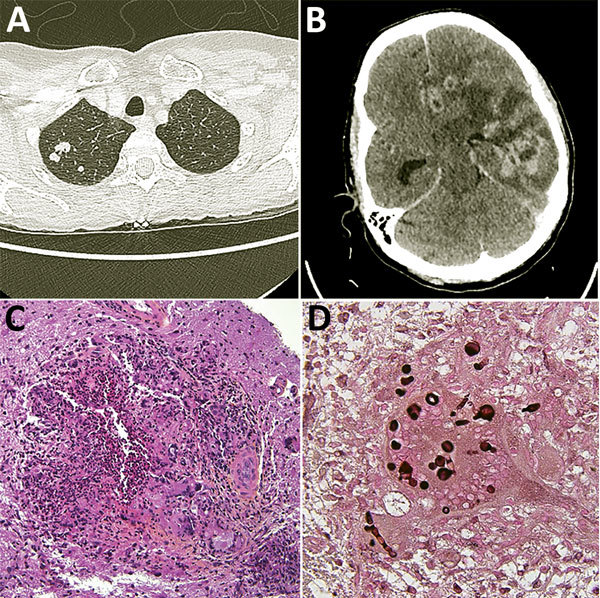
Diagnostic testing of a 52-year-old woman from France living in Mali who had *Nannizziopsis* spp. fungal infection. A) Thoracic-abdominal-pelvic scan shows pseudo-nodular lesions in the apex of the right lung, of which one is excavated. B) Cerebral computed tomography scan shows contrast enhancement on several hemispheric nodules on the left and in frontal, parietal, and temporal regions, responsible for large surrounding edema and compression of the left lateral ventricle. The median line is deviated to the right with a subfalcorial herniation. C) Hematoxylin-eosin-saffron stain of brain biopsy containing mononuclear inflammatory infiltrates; giant cell granulomas; histiocytes, sometimes with an epithelioid appearance; and neutrophils (original magnification ×200). D) Grocott stain showing thick bulbous mycelial filaments in the cytoplasm of certain giant cells/histiocytes (original magnification ×600). Round shapes correspond to cross-sections of bulbous territories.

## Conclusions

The molds of the *N. vriesii* complex (*Chrysosporium*-like anamorph, CANV) are members of the *Nannizziopsis* genus (*Onygenales*, *Eurotiomycetidae*, *Eurotiomycetes*, *Ascomycota*). CANV includes the keratinophilic species, which causes skin and fatal disseminated infections in reptiles (*3**,**4*). There is no documented evidence of zoophilic species involvement in human infections, but 3 other CANV species have been recovered from human samples (Table). Of the 5 previous cases of *Nannizziopsis* infections, 3 involved HIV patients. One of our patients was seropositive for HIV and the other had a T-cell prolymphocytic leukemia, which suggests that lymphopenia could be a key risk factor. All patients with reported *N. obscura* infection had recently traveled in Africa. Results for case-patient 1 showed that the fungus grows in blood cultures and thus has high potential for dissemination. Case-patient 2 had pulmonary lesions, but BAL cultures were rapidly invaded by a *Penicillium* fungus*.* Although we could not detect *Nannizziopsis* in BAL, the lesion evolved favorably after antifungal therapy. Because *Nannizziopsis* spp. are keratinophilic molds, we looked for cutaneous lesions. Both case-patients had recently developed cutaneous or nail lesions during their time in Africa, but we did not detect *Nannizziopsis* from these lesions. These molds have not been isolated in our laboratory in other kinds of samples (clinical or environmental).

CSF cultures from both our case-patients and ascites cultures from case-patient 1 were negative, but panfungal PCR successfully detected molds (*6*). Data on biomarkers are scarce. *N. infrequens* cross-reacts with the *Histoplasma* AccuProbe test and *N. hominis* with the *Blastomyces* AccuProbe test (Hologic, San Diego, CA, USA) (*5*). In case-patient 2, β-D-glucan was positive in CSF and serum but galactomannan antigen was not. *N. obscura* seems to be sensitive to most antifungal agents (*2*).

These observations show how difficult this infection is to detect, which could explain why so few cases of human infections have been reported. However, the diagnosis of these 2 cases since 2015 suggests that the prevalence of *Nannizziopsis* infections may be underestimated.

Technical Appendix Additional information about human infections caused by *Nannizziopsis* molds.
