# Supplementary material for: Invasive Infections Caused by Nannizziopsis spp. Molds in Immunocompromised Patients
Source: Emerg Infect Dis. 2018 Mar;24(3):549–52. doi: 10.3201/eid2403.170772 (PMC5823334; doi:10.3201/eid2403.170772)
Supplement: Technical Appendix — Additional information about human infections caused by Nannizziopsis molds. [file 17-0772-Techapp-s1.pdf]

Article DOI: https://doi.org/10.3201/eid2403.170772

Invasive Infections Caused by *Nannizziopsis* spp. Molds in Immunocompromised Patients

Technical Appendix

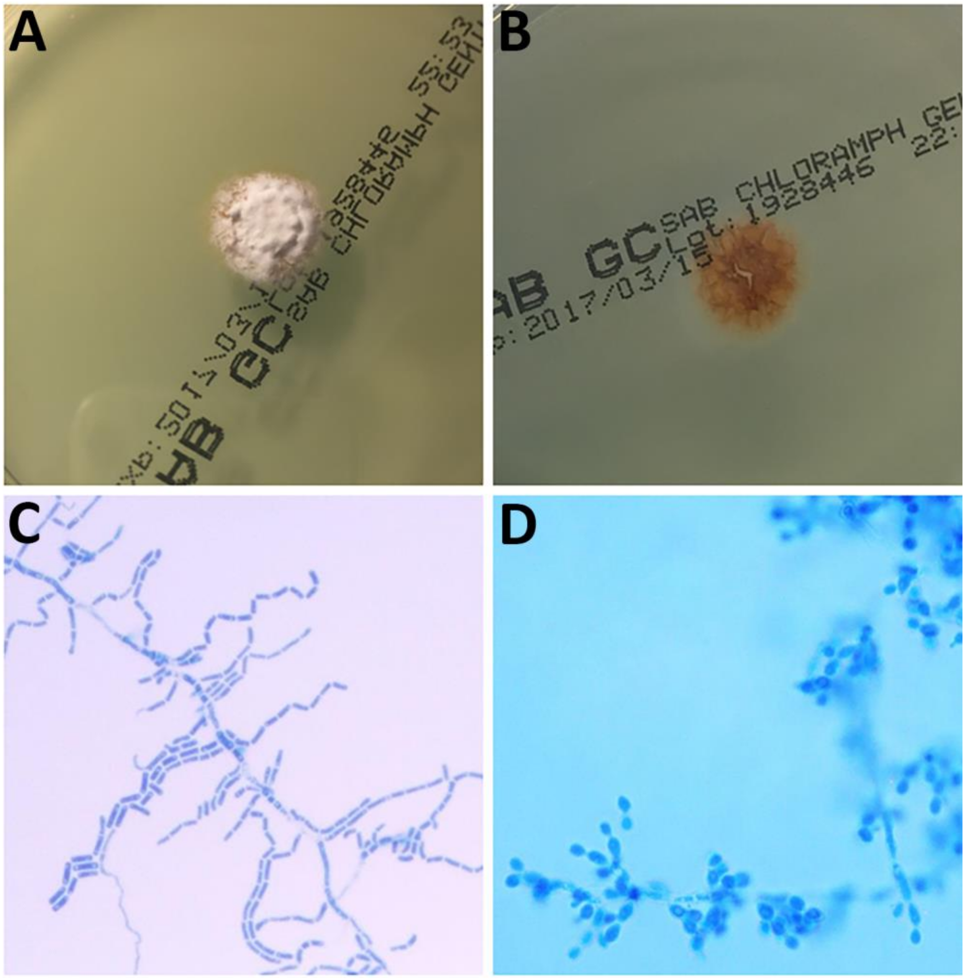

Technical Appendix Figure 1. A) *Nannizziopsis obscura* forming white and thinly cottony mold colonies on Sabouraud medium incubated at 35°C, viewed from the front. B) *N. obscura* specimen viewed from the back. C) Undulate hyphae with chains of adjacent arthroconidia. D) Two-celled specimen of undulate hyphae.

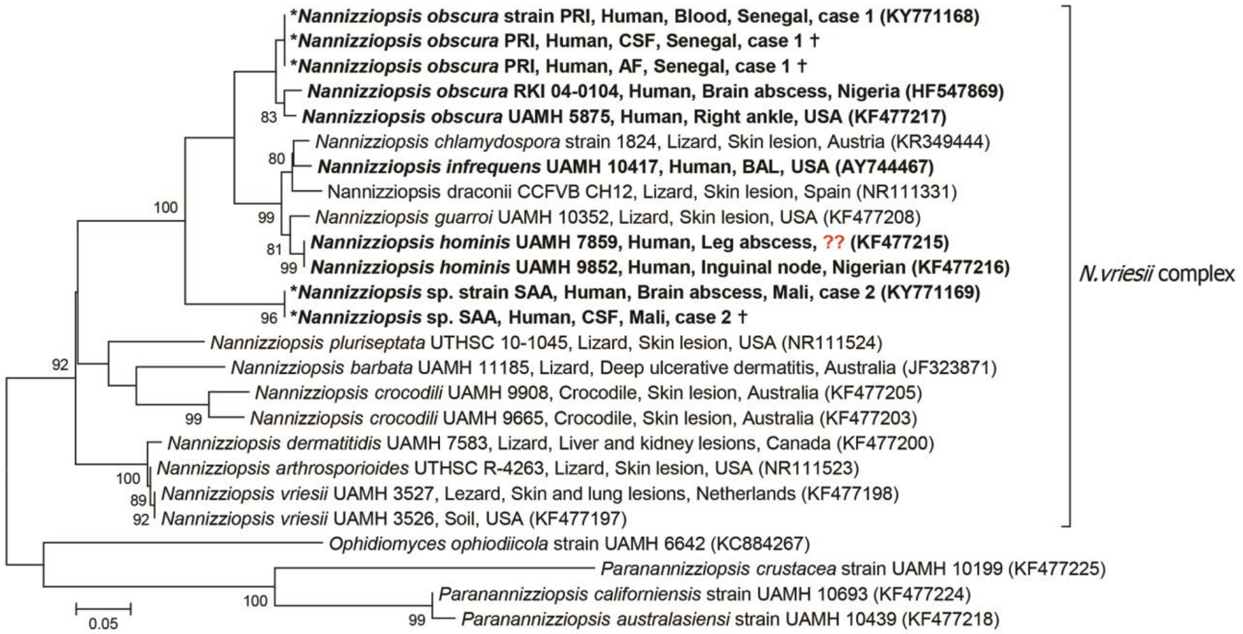

Technical Appendix Figure 2. Molecular phylogenetic analysis of ITS region (from ITS5 to ITS4 primers hybridization region) of *Nannizziopsis vriesii* complex strains inferred by the maximum likelihood method based on the Tamura-Nei model with a discrete gamma distribution (1,000 replicates). Strains from human infections appear in bold. *, strains from our study. †, sequences obtained from ITS4/ITS7 PCR on samples.
